# Clinical Scoring for Prediction of Acute Kidney Injury in Patients with Acute ST-Segment Elevation Myocardial Infarction after Emergency Primary Percutaneous Coronary Intervention

**DOI:** 10.3390/jcm10153402

**Published:** 2021-07-30

**Authors:** Akaphol Kaladee, Phichayut Phinyo, Thamarath Chantadansuwan, Jayanton Patumanond, Boonying Siribumrungwong

**Affiliations:** 1 School of Health Science, Sukhothai Thammathirat Open University, Nonthaburi 11120, Thailand; akaphol.ka@gmail.com; 2 Department of Family Medicine, Faculty of Medicine, Chiang Mai University, Chiang Mai 50200, Thailand; 3 Center for Clinical Epidemiology and Clinical Statistics, Faculty of Medicine, Chiang Mai University, Chiang Mai 50200, Thailand; jpatumanond@gmail.com; 4 Musculoskeletal Science and Translational Research (MSTR) Center, Chiang Mai University, Chiang Mai 50200, Thailand; 5 Cardiology Department, Central Chest Institute, Nonthaburi 11000, Thailand; dansuwan_t@yahoo.co.th; 6 Department of Surgery, Faculty of Medicine, Thammasat University, Pathum Thani 10120, Thailand; boonying22@gmail.com

**Keywords:** risk assessment, ST-elevation myocardial infarction, coronary angiography, percutaneous coronary intervention, kidney diseases

## Abstract

Acute kidney injury (AKI) after a coronary intervention is common in patients with ST-segment elevation myocardial infarction (STEMI) and is associated with significant morbidity and mortality. Several scores have been developed to predict post-procedural AKI over the years. However, the AKI definitions have also evolved, which causes the definitions used in the past to be obsolete. We aimed to develop a prediction score for AKI in patients with STEMI requiring emergency primary percutaneous coronary intervention (pPCI). This study was based on a retrospective cohort of Thai patients with STEMI who underwent pPCI at the Central Chest Institute of Thailand from December 2014 to September 2019. AKI was defined as an increase in serum creatinine of at least 0.3 mg/dL from baseline within 48 h after pPCI. Logistic regression was used for modeling. A total of 1617 patients were included. Of these, 195 patients had AKI (12.1%). Eight significant predictors were identified: age, baseline creatinine, left ventricular ejection fraction (LVEF) < 40%, multi-vessel pPCI, treated with thrombus aspiration, inserted intra-aortic balloon pump (IABP), pre- and intra-procedural cardiogenic shock, and congestive heart failure. The score showed an area under the receiver operating characteristic curve of 0.78 (95% CI 0.75, 0.82) and was well-calibrated. The pPCI-AKI score showed an acceptable predictive performance and was potentially useful to help interventionists stratify the patients and provide optimal preventive management.

## 1. Introduction

Acute coronary syndrome (ACS) is a serious, life-threatening condition and an important health issue in the modern world. ACS covers a range of clinical spectrum and severity, ranging from unstable angina (UA), non-ST-segment elevation myocardial infarction (NSTEMI), and ST-segment elevation myocardial infarction (STEMI) [1]. For patients with STEMI, a timely reperfusion therapy with primary percutaneous coronary intervention (pPCI) is recommended to improve clinical outcomes after the event [2]. However, this life-saving intervention often gives rise to clinically important complications, such as acute kidney injury (AKI). For both patients with STEMI and NSTEMI, AKI was highly prevalent and was associated with an increase in both short- and long-term morbidity and mortality [3]. The incidence in patients with STEMI varied widely across different regions from 8.6% to 55% [4,5,6,7,8,9,10,11,12,13,14,15,16,17,18]. A previous study from Thailand reported an AKI incidence at 19.8% [19]. Multiple factors are attributed to the differences in AKI incidence, including the use of different diagnostic criteria for AKI, such as RIFLE, AKIN, and KDIGO [20,21,22,23].

Although the definite intervention for AKI after a coronary intervention is not yet settled and a general preventive strategy is usually applied in all patients, individual clinical risk assessment is still essential for risk communication. Several clinical prediction rules for AKI prediction were developed over the years, such as Mehran’s risk score (MRS) [24], Marenzi risk score [14], Ghani and Tohamy, (2009) [25], Tziakas et al., (2013) [26], AGEF score [27], Abusaada, Yuan, Sabzwari, Butt, and Maqsood (2017) [28], and UT-AKI [29]. As several clinical and methodological heterogeneity were identified among the reported scoring systems, the selection of the most appropriate scoring system to be applied in real world clinical settings could not be based entirely on the reported apparent discriminative ability. Ideally, all scoring systems should be externally validated in a target population to confirm the robustness of their performance before being considered for clinical implementation [30]. However, a decrease in the score performance was common, especially when the validation was conducted in different geographical regions with a different population case-mix.

Recently, a practical risk stratification score for AKI, the Central Chest Institute of Thailand (CCIT) score, was developed from a cohort of Thai patients with STEMI [19]. The CCIT score contains only three simple predictors and carried good predictive ability. In addition, as the CCIT score was developed from an entire cohort of Thai population, the generalizability of the score to the Thai population was high. However, the CCIT score uses the same definition for the predicted endpoint as the MRS [24], which is, nowadays, considered irrelevant, as the current guideline had raised the importance of AKI and AKI definitions had been evolved [23,31]. Moreover, the discriminative ability of the original CCIT score might be overfitted as the score was developed from a rather small cohort of patients. Our study aimed to develop a new clinical prediction score for AKI in patients with STEMI who underwent pPCI based on the original framework of the CCIT score.

## 2. Materials and Methods

### 2.1. Design and Setting

Prognosis research with prediction score development was conducted based on a retrospective observational cohort of patients diagnosed with STEMI who underwent pPCI at the Central Chest Institute of Thailand from December 2014 to September 2019. The Central Chest Institute of Thailand, or CCIT, is a tertiary care hospital with 333 in-hospital beds specializing in cardiopulmonary diseases. The Human Research Ethics Committee of Thammasat University No.1, Faculty of Medicine [COA 212/2019 MTU-EC-ES-6-173/62] and the Human Research Ethics Committee of the Central Chest Institute, Department of Medical Services, Ministry of Public Health of Thailand [COA 035/2563] approved the study protocol.

### 2.2. Patients

Adult patients diagnosed with acute STEMI who were treated with pPCI during the study period were included. We excluded patients with end-stage renal disease (ESRD) who had an estimated glomerular filtration rate (eGFR) of less than 15 mL/min/1.73 m^2^ or patients who were treated with hemodialysis or peritoneal dialysis, patients who were exposed to contrast media within the past 7 days, patients who did not survive pPCI, and patients who did not have serum creatinine (SCr) results within 48 h after pPCI.

### 2.3. Definition of AKI

In this study, we defined the occurrence of AKI based on the definition by KDIGO serum creatinine criteria [23], which was an increase in serum creatinine of at least 0.3 mg/dL from baseline within 48 h after pPCI.

### 2.4. Data Collection and Predictors

We collected the baseline data on age, gender, anthropometric measurements (i.e., weight, height, body mass index, and body surface area), presence of comorbidity (i.e., hypertension, diabetes mellitus, hypercholesterolemia, anemia, previous MI, previous stroke or TIA, peripheral artery disease (PAD), and pre-existing renal disease), past medical history and previous medication used for other diseases, and history of substance use. Anemia was defined according to the WHO’s definition [32]. Pre-existing renal disease was defined as patients with baseline serum creatinine >1.5 md/dL or eGFR less than 60 mL/min/1.73 m^2^) [33]. eGFR was calculated with the Chronic Kidney Disease Epidemiology Collaboration (CKD-EPI) formula [34]. Data on initial vital signs (e.g., systolic and diastolic blood pressure), relevant laboratory investigations (e.g., Troponin-T and serum creatinine) at initial presentation, wall of infarction from electrocardiogram (ECG), echocardiographic reports, angiographic findings, type of pPCI performed, type and volume of contrast media used, intra-aortic balloon pump (IABP) insertion, pre- and intra-procedural hypotension, cardiogenic shock, congestive heart failure (CHF), total ischemic time, and treatment outcomes (e.g., hemodialysis and length of hospital stay) were also collected. Supplementary Table S1 lists the definitions of the following prognostic determinants in detail: previous medication used, pre- and intra-procedural hypotension, cardiogenic shock, CHF, and total ischemic time.

In our institution, blood samples for initial laboratory investigations, including serum creatinine and cardiac markers, would be taken in all patients undergoing pPCI. As the point of prediction of the score was after the patients were transferred back to the cardiac intensive care unit after pPCI, data on all laboratory parameters were available.

### 2.5. Study Size Estimation

As the mean to estimate the adequate study size for developing clinical prediction score was not yet settled, we employed two statistical approaches in estimating the study size. First, we estimated the study size based on the comparison of proportion of potentially significant predictors (i.e., being treated with multi-vessel pPCI) between groups of patients with AKI and without AKI using the previously reported CCIT data [19]. The proportion of patients who were treated with multi-vessel pPCI with AKI were 0.1 and without AKI were 0.04. Given a ratio between AKI and non-AKI was 1:6, a total study size of 1165 patients were required to achieve an 80% statistical power, and a two-sided alpha error of 0.05. Second, we followed the general suggestion of the TRIPOD statement that at least 10–20 events are needed for one predictor variable in the logistic model [35]. As we anticipated that our model would include up to 10 predictor variables, a total of 200 AKI events were required. Thus, based on the reported incidence of AKI in STEMI patients at 0.14 [19], we planned to include at least 1428 patients.

### 2.6. Statistical Analysis and Score Development

We described continuous variables with mean and standard deviation. Frequency and percentage were used for categorical variables. Fisher’s exact probability test was used to compare categorical variables, whereas an independent *t*-test was used for continuous variables. To examine the discriminative ability of potential predictors, we estimated the area under the receiver operating characteristic (AuROC) curve for each predictor. All statistical analyses were performed using Stata 16 (StataCorp, College Station, TX, USA). A *p*-value less than 0.05 was considered a statistically significant difference.

The selection of potential predictors for the prognostic score was based on the statistical significance of the univariable analysis. All predictors with a univariable *p*-value less than 0.20 were included in a multivariable logistic model [36]. Then, predictors with a *p*-value more than 0.05 and an odds ratio close to 1.0 were sequentially removed from the model in a backward fashion. Each predictor in the final model was assigned with a weighted score that was calculated by dividing the logit coefficient of each predictor with the lowest coefficient in the model. Therefore, the predictor with the lowest value of the logit coefficient will be scored one. The products of the division were then rounded up to whole numbers. The sum of the total score for each individual in the dataset was then used to test for the predictive performance. The score performance was evaluated in terms of model discrimination and calibration. The discriminative ability of the score was based on AuROC. The score calibration was based on the score calibration plot and the Hosmer-Lemeshow goodness-of-fit test. The score calibration plot was created by contrasting the sum of the total score with the observed proportion of AKI within each score stratum. Decision curve analysis was employed to examine the clinical utility of the score [37]. The net benefit (NB) of the newly developed score was presented with the NB of other two default strategies of treating all patients (treat all) or not treating any patient (treat none).

For clinical applicability of the score, all the patients in the dataset were grouped into three risk categories: low, moderate, and high risk. The score was categorized into three risk groups rather than two groups to preserve the loss of information from dichotomization and to enable clinicians to prioritize patients for early aggressive intervention better. The score cutoff points were chosen based on the appropriateness of sensitivity and specificity. A higher sensitivity with acceptable specificity (≥50%) was required for the lower cutoff point, whereas a higher specificity with acceptable sensitivity (≥50%) was needed for the higher cutoff point. We calculated a positive likelihood ratio (LHR+) with its corresponding 95% confidence interval (CI) to represent the ability of each score’s category in the prediction of AKI. An LHR+ less than 1 indicates that the odds of AKI are significantly lower than the general population, whereas an LHR+ higher than 1 indicates that the odds of AKI is significantly higher than the general population. We re-examined the discriminative ability of the score categories by observing whether the confidence interval of LHR+ for each score category overlaps one another. To quantify the optimism of the derived score in the prediction of AKI, we performed an internal validation of the score with bootstrap re-sampling procedures.

We performed a comparative validation of the newly developed score with previously reported prediction rules and scoring systems, which were the CCIT score [19], the Mehran’s risk score [24], the Ghani and Tohamy score [25], and the AGEF score [27]. The discriminative ability of each score was compared to the newly developed score using tests of equality of AuROC. Adjustment of multiple comparisons was done with Sidak’s method. We also plotted the smoothened NB of each scoring across a range of threshold probability on decision curves to explore the potential clinical utility.

## 3. Results

**Figure 1:**
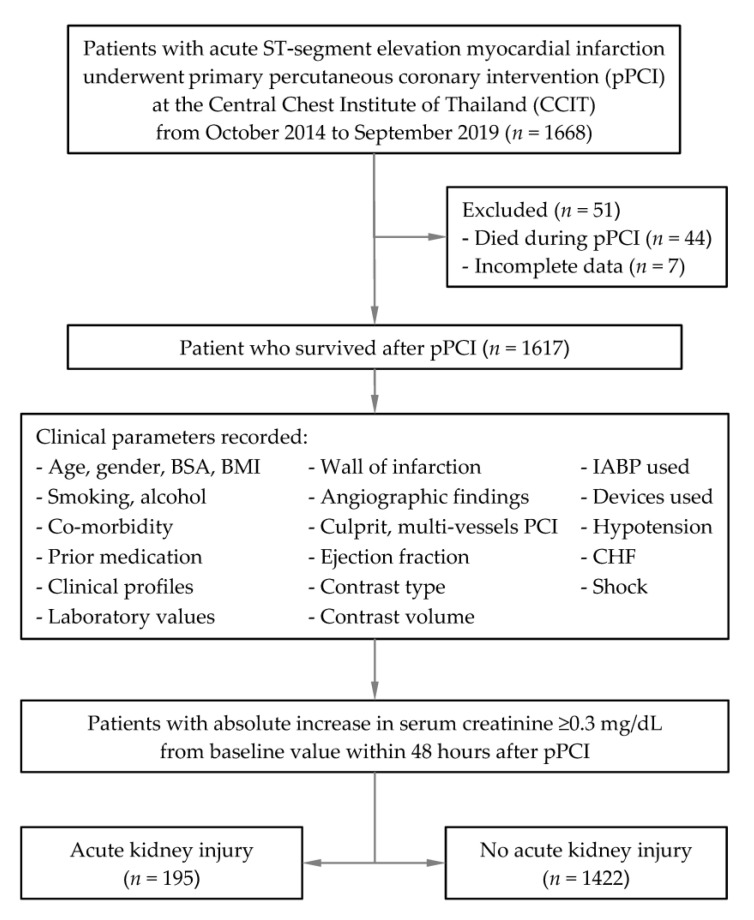
Flow diagram of patient details. Abbreviations: pPCI, primary percutaneous coronary intervention; CCIT, Central Chest Institute of Thailand; BSA, body surface area; BMI, body mass index; IABP, intra-aortic balloon pump; CHF, congestive heart failure.

From October 2014 to September 2019, a total of 1668 adult patients with acute STEMI who underwent pPCI at our institution were included. Of these patients, 51 patients were excluded (Figure 1). From the remaining 1617 patients, there were 195 patients with AKI with an estimated incidence of 12.1% (95% CI 10.5% to 13.8%). Table 1 compares prognostic characteristics, procedural characteristics, angiographic findings, and complications between patients with and without AKI. Supplementary Table S2 shows and compares previous medication used between patients with and without AKI. The average contrast media volume used in this study was 116.0 ± 40.6 mL. Only 71 (4.4%) patients had a contrast media volume per eGFR of more than 3.7. Several factors were identified as significant predictors from univariable analysis and were subsequently included in the multivariable model (Table 1).

After backward elimination of non-significant predictors from the multivariable logistic regression model, the following factors were identified as significant predictors for AKI in STEMI patients: age, baseline creatinine, left ventricular ejection fraction <40%, treated with multi-vessel pPCI, treated with thrombus aspiration, IABP inserted, pre- and intra-procedural cardiogenic shock, and pre- and intra-procedural CHF (Table 2). After the score transformation, the sum of the derived score ranges from 0 to 12.5, with an overall mean score of all patients at 3.2 ± 2.6. The average score differed significantly between patients with and without AKI (5.8 ± 3.1 vs. 2.8 ± 2.2, *p* < 0.001). Two cutoff points were identified to classify all included patients into three risk categories: low, moderate, and high risk. Supplementary Table S3 shows the sensitivity and specificity of each score cutoff point. The lower cutoff point of the score was set at ≥3 with a sensitivity of 81.5% (95% CI 75.4, 86.7) and a specificity of 56.9% (95% CI 54.3, 59.5). The higher cutoff point was set at ≥5 with a sensitivity of 55.9% (95% CI 48.6, 63.0) and a specificity of 82.9% (95% CI 80.9, 84.8).

**Figure 2:**
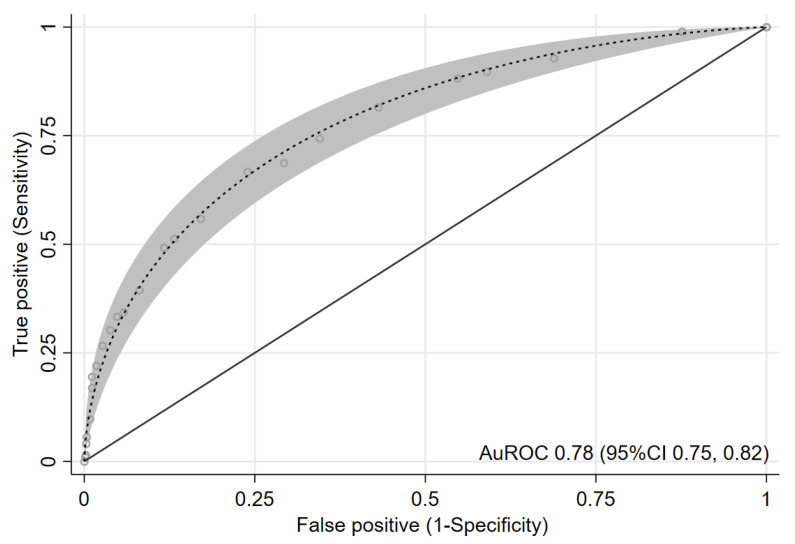
ROC curve (parametric) and 95% confidence band for prediction of AKI by the derived score. Abbreviations: AKI, acute kidney injury; AuROC, area under the receiver operating characteristic curve; CI, confidence interval.

**Figure 3:**
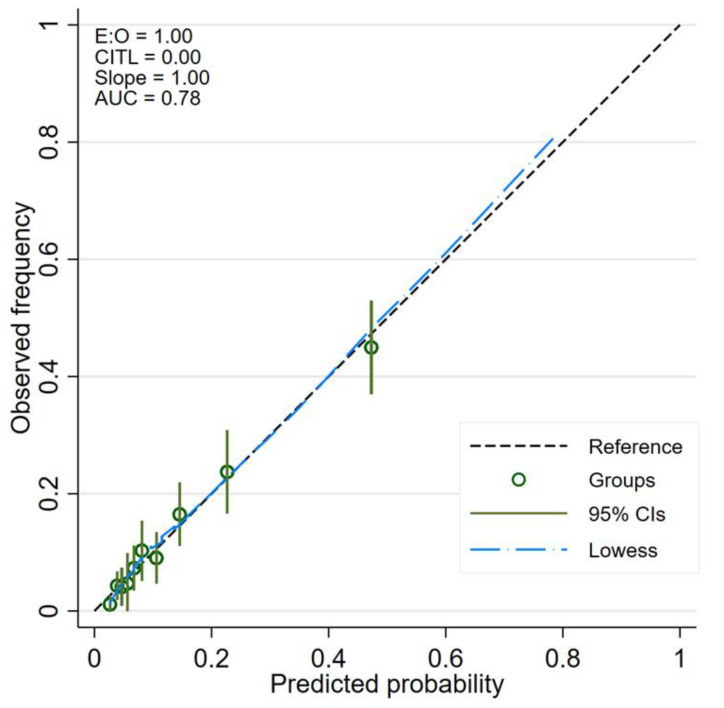
Calibration plot between the score-predicted probability of AKI and observed AKI. Abbreviations: AUC, area under the curve; CIs, confidence intervals; CITL, calibration-in-the-large.

**Figure 4:**
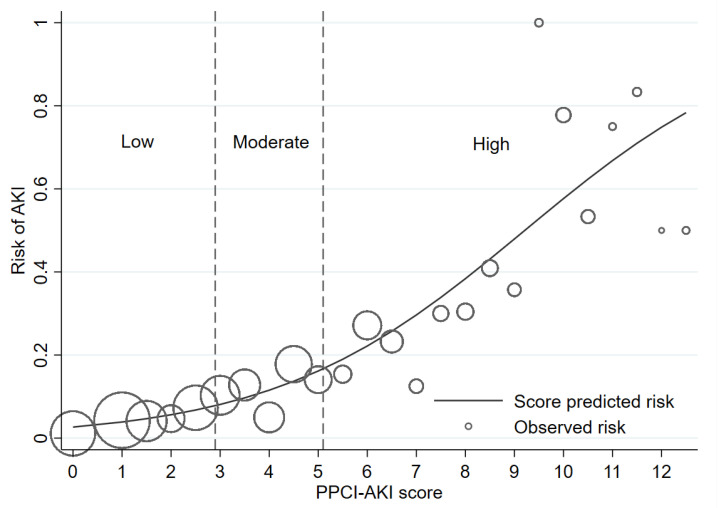
Predicted risk AKI by score (solid line) and observed risk of AKI (hollow circles). Size of circles depicts relative number of patients in each score. Abbreviations: AKI, acute kidney injury.

We present the prevalence of AKI and the LHR+ for each category in Table 3. The confidence interval of LHR+ for each risk category did not overlap one another. The score showed an acceptable to good ability to discriminate between patients with and without AKI based on an apparent AuROC at 0.78 (95% CI 0.75, 0.82) (Figure 2). After internal validation with the bootstrap procedure, the test AuROC dropped minimally to 0.75 (95% CI 0.72, 0.79). In terms of score calibration, the Hosmer-Lemeshow goodness-of-fit statistics revealed non-significant result (no statistical evidence of lack-of-fit) (*p* = 0.829), indicating an almost perfect fit of the model to the observed data. The score calibration plot also showed good agreement between the AKI risk score and the observed risk of AKI (Figure 3). The decision curve analysis showed potential clinical usefulness of the AKI risk score, as the NB of the AKI risk score was higher than the two default strategies of treating all patients as AKI or not treating any patients as AKI across the whole range of threshold probability (Figure 4). The score is presented as an online web application for clinical applicability, which can be accessed through the following link: https://www.ppciakiscore.com (accessed on 26 July 2021).

**Figure 5:**
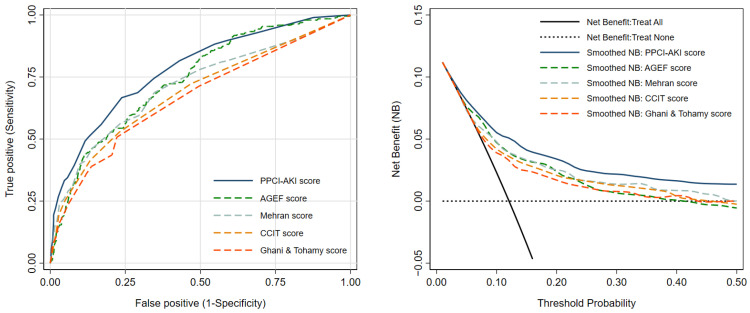
Comparative validation of the PPCI-AKI score to previously reported scoring systems in terms of discriminative ability (left) and clinical utility (right). Abbreviations: NB, net benefit; AGEF score, age, glomerular filtration rate, ejection fraction score; CCIT score, a score by central chest institute of Thailand.

For comparative validation in terms of discriminative performance, the AuROC of the PPCI-AKI score at 0.78 (95% CI 0.75, 0.82) was significantly superior to the AGEF score at 0.74 (95% CI 0.70, 0.78) (*p*-value = 0.0146), the Mehran’s risk score at 0.71 (95% CI 0.67, 0.75) (*p*-value < 0.001), the CCIT score at 0.69 (95% CI 0.65, 0.73) (*p*-value < 0.001), and the Ghani and Tohamy score at 0.67 (95% CI 0.63, 0.71) (*p*-value < 0.001) (Figure 5). In addition, the PPCI-AKI score also showed a higher value of NB than all other scoring systems across the range of threshold probability (Figure 5).

Patients with AKI were likely to have higher serum creatinine levels and lower eGFR than patients without AKI at 48 h and 3 months after pPCI (Table 4). Moreover, patients with AKI after pPCI had a significantly longer length of hospital stay (10.4 ± 13.0 vs. 6.9 ± 6.3, *p* < 0.001), a higher proportion of referral for hemodialysis (0.021 vs. 0.001, *p*-value = 0.003), and a higher proportion of in-hospital death (0.174 vs. 0.028, *p* < 0.001) than patients without AKI after pPCI.

## 4. Discussion

In this study, we developed a new clinical risk score to predict AKI in patients with acute STEMI after pPCI: the PPCI-AKI score. The development of the PPCI-AKI score was based on the previously developed CCIT score with the addition of several clinically relevant predictors. We also increase the size of the derivation dataset to prevent overfitting issues. The score includes eight routinely assessable predictors, which were the age of the patient, baseline serum creatinine, left ventricular ejection fraction upon arrival, type of pPCI treated, the requirement of thrombus aspiration, the requirement of IABP insertion, pre- and intra-procedural cardiogenic shock, and pre- and intra-procedural CHF. The score showed acceptable discriminative performance with good calibration and was potentially useful for clinical use.

The AKI incidence in previous clinical prediction studies varied from 8.6% up to 55% [4,5,6,7,8,9,10,11,12,13,14,15,16,17,18]. In our study, the AKI incidence was estimated at 12.1%, which was within the range reported in the literature. The use of different AKI definitions in each study undoubtedly explains the heterogeneity in AKI incidence across studies. Three studies used the KDIGO AKI definition as ours. However, only one had a similar AKI incidence to our study at 10.6% [7]. The other two studies reported higher AKI incidence at 23.0% to 23.7% [13,17]. Thus, other factors may also attribute to the differences in the AKI incidence, such as the difference in the study samples, the geographical location, the criteria used for the changes of serum creatinine, and the timing of endpoint measurements.

Our study developed an AKI risk prediction score specifically for acute STEMI patients requiring emergency pPCI, which was in contrast to the MRS that includes only patients who underwent elective pPCI [24], an entirely different patient domain. Some studies included a mixed population of patients, both emergency pPCI and elective pPCI [25,26,28]. The proportion of patients who underwent elective pPCI in these studies would affect the generalizability of their results to the emergency pPCI population, as the incidence of AKI largely differed between emergency and elective pPCI populations [26]. To date, only four clinical prediction scores were developed entirely from a sample of STEMI patients who underwent emergency pPCI [14,19,27,29], including the CCIT score. Most studies defined AKI based on a traditional criterion of contrast-induced nephropathy (i.e., an increase in serum creatinine ≥0.5 mg/dL or ≥25% from baseline value 48 or 72 h after pPCI [14,19,24,25,26,27]. Some studies defined AKI according to RIFLE and AKIN criteria [28,29]. Our study defined AKI according to the KDIGO criteria, which is a current standard criterion. Regardless of methodological differences, most of the clinical prediction scores, including the newly-derived PPCI-AKI score, showed consistent performance at an acceptable level (0.7 ≤ AuROC < 0.8) [14,19,24,25,26,27,28,29].

Eight routinely evaluated predictors were included in the PPCI-AKI score. Some of the predictors were supported by previous evidence and included in other scoring systems, such as older age [14,24,27], higher baseline serum creatinine [25], LVEF <40% [19,27,29], multi-vessel pPCI [19,25], cardiogenic shock at presentation [25], congestive heart failure at presentation [24,28], and insertion of IABP. Both older age and high baseline serum creatinine indicate impaired renal function, which can be further aggravated by the exposure to contrast media during the procedure. The remaining features reflect the presence of hemodynamic dysfunction, which ultimately results in poor renal blood flow and AKI. Thrombus aspiration was identified as a new predictor for AKI in patients requiring emergency pPCI in our study. We hypothesized that the use of a thrombus aspiration catheter was a surrogate or an indicator of severe occlusion of the coronary artery by thrombus, which leads to prolonged duration of cardiac ischemia and total ischemic time [38,39]. Previously, routine aspiration thrombectomy before pPCI was recommended by STEMI guidelines with class IIA recommendation in 2011/2013. However, it was later downgraded to class IIB in 2015 and class III in 2018 [40]. These modifications were according to the results of the TOTAL trial [41], which found that routine thrombus aspiration did not reduce cardiovascular mortality but increased the incidence of stroke at 30 days. Our study data were collected after 2013. The choice of aspiration thrombectomy was up to the interventionists and was not routinely performed in all cases of emergency pPCI.

Due to the differences in the definition of predictors and the occurrence of AKI, a variety of predictors was included in each scoring system. Some predictors reported by other studies were not identified and included as predictors in the PPCI-AKI score, which were a pre-existing renal disease, diabetes mellitus, anemia, estimated GFR, and contrast media volume. The type of contrast media used during pPCI was not identified as an independent predictor for AKI in this study. One reason was the use of Iso-osmolar contrast media, Iodixanol-320, at our institution during the study period. These types of contrast media carry fewer side effects than the low-osmolar or the high-osmolar contrast media [42]. The volume of contrast media used was also not a predictor for AKI, which was in contrast to three previous studies that identified the significance of contrast media volume as an independent risk factor for AKI [14,24,26]. However, this can be explained by the less contrast media volume used in our center than in the previous studies by Mehran et al. [24] and Marenzi et al. [14]. For this reason, an approach to minimize contrast media volume during the procedure (i.e., ultra-low coronary angiography and zero-contrast PCI) should be adopted in practice to prevent post-procedural AKI [43].

Our study focused on AKI occurrence within 48 h after pPCI using predictors available immediately after pPCI. Therefore, relevant clinical patient data after the point of prediction was not considered in making the prediction. It is likely that the patient’s condition and clinical progression during admission would also have value in predicting AKI and mortality. A recent study proposed the potential role of declining hemoglobin levels during hospitalization in predicting in patients with acute coronary syndrome. Although the endpoint used in the study was not the occurrence of AKI, hemoglobin drop during admission has a strong pathophysiologic background to negatively affects the patient’s renal function and causes AKI following pPCI [44]. Future studies should examine the possibility of incorporating hemoglobin declination during admission as a dynamic predictor for AKI and mortality after pPCI.

According to the comparative validation performed in this study, the PPCI-AKI score was superior to the other four scoring systems in terms of both discriminative performance and NB over the range of threshold probability. Table 5 shows and compares methodological characteristics of all the scores validated in this study. Of the four scoring systems, the AGEF score was the only scoring that did not include intra-procedural factors [27]. However, its discriminative ability was closest to that of the PPCI-AKI score, which needs five more intra-procedural factors. The PPCI-AKI score included factors similar to the AGEF score: age groups, LVEF, and factor reflecting baseline renal function (baseline creatinine for PPCI-AKI score and eGFR for AGEF score). This finding supports the strength of association and the predictive ability of these three pre-procedural factors in predicting post-procedural AKI. Nonetheless, the inclusion of intra-procedural factors in the PPCI-AKI score significantly enhanced its discriminative ability. The drop in performance of the other scoring systems might be the results of AKI definitions during score development and the differences in the predictor-outcome associations. Still, it is important to note that this comparative validation might be subjected to bias as the PPCI-AKI score was developed from this dataset. Thus, the performance measures of the PPCI-AKI score were likely to be overestimated. Further comparative validation should be conducted in an entirely external dataset.

The newly developed PPCI-AKI score carries potential utility for clinical practice. As the data on all predictors would routinely be available after the PCI was done, the PPCI-AKI score can be used to predict the patients’ risk for AKI upon their arrival to the cardiac critical care unit (CCU). The score predictions can help interventionists properly stratify patients requiring emergency pPCI into risk groups and manage accordingly. Some preventive measures are recommended to all patients regardless of the risk prediction, such as avoiding nephrotoxic drugs [25,42], using iso-osmolar contrast media at minimum volume possible, and continuous monitoring of delayed AKI. For patients with a low risk of AKI, we suggested adequate oral or intravenous hydration as appropriate. Infusion of normal saline solution in the appropriate amount (approximately 1 mL/kg/hour) over 12 h after pPCI is suggested for patients at moderate to high risk of AKI. Special attention should be paid to patients with high risk. Mean arterial pressure should be monitored and maintained above 65 mmHg. We suggested following serum creatinine at three months for surveillance of persistent renal damage. In addition, the PPCI-AKI score might be potentially useful during the coronavirus 19 (COVID-19) pandemic, as tailored intervention could be effectively provided according to each patient’s risk for AKI, which may reduce the occurrence or, at least, reduce the severity of AKI. With appropriate management, the proportion of patients who required renal replacement therapy (RRT) might decrease. Therefore, dialysis could be efficiently allocated to patients in need, and non-dialytic management could be offered to patients with AKI stage I or II, especially when dialysis resources are limited [45].

Our study carried both strengths and limitations. In terms of strengths, first, the study size was larger compared to the previous one [19]. Second, the PPCI-AKI score was derived using the latest definitions of AKI according to KDIGO [23]. Third, we followed a methodological and statistical standard in the derivation of the prognostic score. All factors included in the model were pre-selected based on previous clinical evidence, clinical experience, and statistical significance. Predictors incorporated in the final model were readily available in practice and supported by strong theoretical background [46]. Moreover, one novel predictor of post-procedural AKI was identified in our study: thrombus aspiration. Fourth, we evaluated the model performance in multiple dimensions, including the clinical usefulness based on the decision curve analysis, which had never been performed in any previously developed scoring systems before.

However, there are five main limitations to our study. First, the data used were retrospectively collected and might be subject to bias and missing data. However, as all clinical data relevant to the patients and the pPCI were routinely collected into a standardized record form, the proportion of missing data was low, at only 3.1%. Thus, the complete case analysis of the data still carried adequate statistical to develop the score. Second, our study limited the point of AKI prediction to only 48 h. Any AKI event beyond this time point was not considered in the analysis, which may underestimate AKI incidence. Nonetheless, most clinical prediction rules in this context still focused on AKI occurrence at 48 h [14,19,24,25,26,28], and only some used other time points [27,29]. Despite the differences in the timing of the predicted endpoint, similar predictors were identified and included in the model, such as age, LVEF, IABP, and factors reflecting renal function (e.g., eGFR). Third, some potential predictors for AKI after coronary intervention were not examined in this study, such as vascular access site, where femoral access site was found to be associated with higher incidence of AKI than radial access [47]. Future studies should consider incorporating vascular access sites during derivation of the prediction model or examining the added predictive value of this factor to the PPCI-AKI score. Fourth, the PPCI-AKI score requires both pre-procedural and intra-procedural factors to predict AKI risk. Therefore, the PPCI-AKI score could not be used until after pPCI. As a result, initiation of preventive strategies is not possible before or during the procedure. However, as intra-procedural factors were consistently identified as independent predictors in the literature and are included in most prediction rules, we believe that they still carry valuable prognostic value. Not including these important intra-procedural factors within the score, the PPCI-AKI score might lose the trust of clinicians, which is a common barrier to the use of prediction rules in practice [48]. Finally, the study was based on a single tertiary care center with specific characteristics of the population. Thus, the generalizability of the PPCI-AKI score to other clinical settings, or other population with different characteristics, must be confirmed by a prospective external validation study before being implemented in practice. Moreover, future studies should address the ability and clinical utility of the PPCI-AKI score to other clinically relevant endpoints, such as chronic dialysis treatment and mortality.

## 5. Conclusions

Eight routinely available predictors in the PPCI-AKI score showed acceptable discriminative ability and calibration in the prediction of AKI in Thai patients with acute STEMI requiring emergency pPCI. The score might be applied to help interventionists stratify the patients into risk groups for an appropriate management as well as provide effective risk communication to the patients and their families. Nonetheless, an external validation study of the PPCI-AKI score is warranted before it can be implemented in clinical practice.

## Figures and Tables

**Table 1:** Baseline characteristics, pathology, pathophysiology, procedure, findings, and complication in patients with or without AKI.

Clinical Characteristics	AKI	No AKI	*p*-Value	AuROC (95% CI)
	(*n* = 195)	(*n* = 1422)		
Female	60 (30.8)	332 (23.4)	0.026	0.53 (0.51–0.56)
Age (year)	67.0 ± 13.2	59.9 ± 12.3	<0.001	0.66 (0.62–0.70)
Weight (kg)	62.4 ± 11.9	66.3 ± 13.4	<0.001	0.58 (0.53–0.62)
Height (cm)	161.6 ± 8.1	164.2 ± 7.9	<0.001	0.59 (0.54–0.63)
BMI (kg/m^2^)	23.8 ± 3.7	24.5 ± 4.2	0.025	0.55 (0.50–0.59)
Body surface area (m^2^)	1.7 ± 0.2	1.7 ± 0.2	0.044	0.54 (0.50–0.58)
Hypertension	112 (57.4)	672 (47.3)	0.009	0.55 (0.53–0.58)
Diabetes mellitus	48 (24.6)	355 (25.0)	1.000	0.50 (0.47–0.52)
Hypercholesterolemia	79 (40.5)	509 (35.8)	0.205	0.52 (0.50–0.55)
Anemia	80 (41.0)	353 (24.8)	<0.001	0.58 (0.56–0.60)
Previous medications used	50 (25.6)	365 (25.7)	1.000	0.50 (0.48–0.52)
SBP (mmHg)	121.2 ± 31.7	127.5 ± 27.8	0.004	0.56 (0.52–0.61)
DBP (mmHg)	74.6 ± 19.9	79.2 ± 15.9	<0.001	0.58 (0.54–0.63)
Troponin-T (×10^2^)	21.2 ± 28.5	15.4 ± 24.7	0.003	0.58 (0.53–0.62)
Baseline creatinine (mg/dL)	1.2 ± 0.6	1.0 ± 0.4	<0.001	0.69 (0.65–0.74)
Wall of infarction from ECG			0.691	0.52 (0.48–0.56)
Anterior wall	102 (52.3)	696 (49.0)		
Inferior wall	84 (43.1)	656 (46.1)		
Others	9 (4.6)	70 (4.9)		
LVEF			<0.001	0.61 (0.58–0.63)
<40%	80 (41.0)	278 (19.6)		
≥40%	115 (59.0)	1144 (80.4)		
Angiographic findings			0.001	0.58 (0.55–0.60)
Single vessel disease	46 (23.6)	500 (35.2)		
Double vessels disease	59 (30.3)	432 (30.4)		
Triple vessels disease	90 (46.2)	490 (34.5)		
Left main coronary artery			0.001	0.54 (0.51–0.56)
<50% stenosis	166 (85.1)	1321 (92.9)		
≥50% stenosis	29 (14.9)	101 (7.1)		
Type of pPCI			0.003	0.52 (0.50–0.55)
Culprit vessel pPCI treated	180 (92.3)	1380 (97.1)		
Multi-vessel pPCI treated	15 (7.7)	42 (2.9)		
Contrast type			0.035	0.52 (0.50–0.55)
Iodixanol-320	1 (0.5)	7 (0.5)		
Ioversol-350	18 (9.2)	68 (4.8)		
Iopromide-370	176 (90.3)	1347 (94.7)		
Contrast volume (mL)	115.6 ± 38.5	116.0 ± 41.0	0.899	0.50 (0.46–0.55)
Thrombus aspiration treated	130 (66.7)	825 (58.0)	0.024	0.54 (0.52–0.57)
IABP treated	61 (31.3)	92 (6.5)	<0.001	0.62 (0.60–0.65)
Pre- and intra-procedural hypotension	75 (38.5)	233 (16.4)	<0.001	0.61 (0.59–0.63)
Pre- and intra-procedural cardiogenic shock	47 (24.1)	69 (4.9)	<0.001	0.60 (0.57–0.62)
Pre- and intra-procedural CHF	23 (11.8)	49 (3.5)	<0.001	0.54 (0.52–0.57)
Total ischemic time (minute)	469 ± 283	432 ± 273	0.079	0.55 (0.51–0.59)

Numbers are *n* (%), or mean ± SD Abbreviations: AuROC, area under the receiver operating characteristic curve; BMI, body mass index; CHF, congestive heart failure; DBP, diastolic blood pressure; ECG, electrocardiogram; IABP, intra-aortic balloon pump; LVEF, left ventricular ejection fraction; pPCI, primary percutaneous coronary intervention; SBP, systolic blood pressure.

**Table 2:** Odds Ratio, 95% CI, coefficient (β), and assigned item score of significant predictors under multivariable regression analysis.

Predictors	OR	95% CI	*p*-Value	β	Item Score
Age (years)					
<60	1.00	Reference		0	0
60–75	1.93	1.31–2.85	0.001	0.66	1.5
≥76	3.15	2.01–4.93	<0.001	1.14	3
Baseline creatinine (mg/dL)					
≤1.00	1.00	Reference		0	0
1.01–2.00	2.24	1.57–3.17	<0.001	0.80	2
≥2.01	5.71	2.70–12.06	<0.001	1.74	4.5
LVEF <40%	1.65	1.15–2.38	0.007	0.50	1.5
Multi-vessel pPCI-treated	2.15	1.08–4.31	0.030	0.77	2
Thrombus aspiration-treated	1.46	1.03–2.07	0.033	0.38	1
IABP-treated	2.36	1.43–3.89	0.001	0.86	2
Pre- and intra-procedural cardiogenic shock	2.22	1.29–3.83	0.004	0.80	2
Pre- and intra-procedural CHF	2.15	1.19–3.88	0.011	0.76	2

Abbreviations: β, beta-coefficient; CHF, congestive heart failure; CI, confidence interval; IABP, intra-aortic balloon pump; LVEF, left ventricular ejection fraction; OR, odds ratio; pPCI, primary percutaneous coronary intervention.

**Table 3:** Distribution of AKI, non-AKI, and likelihood ratio of AKI (LHR+) in low-, moderate-, and high-risk categories.

Risk Categories	Score	Prevalence (%)	AKI (*n* = 195)	No AKI (*n* = 1422)	LHR+	95% CI	*p*-Value
Low	<3.0	18.5	36 (4.3)	809 (95.7)	0.32	0.24–0.44	<0.001
Moderate	3.0–5.0	30.2	59 (12.2)	425 (87.8)	1.01	0.81–1.27	0.934
High	>5.0	51.3	100 (34.7)	188 (65.3)	3.88	3.20–4.69	<0.001
Mean ± SD			5.8 ± 3.1	2.8 ± 2.2			<0.001

Numbers are *n* (%) or mean ± SD. Abbreviations: AKI, acute kidney injury; CI, confidence interval; LHR+, positive likelihood ratio; SD, standard deviation.

**Table 4:** Endpoints and prognosis of patient with or without AKI after PCA.

Clinical Endpoints	AKI	No AKI	*p*-Value
	(*n* = 195)	(*n* = 1422)	
**48 h after pPCI**			
Creatinine (mg/dL)	1.8 ± 0.9	1.0 ± 0.3	<0.001
eGFR (mL/min/1.73 m^2^)	41.9 ± 20.0	84.6 ± 22.7	<0.001
3 months after pPCI			
Creatinine (mg/dL)	1.4 ± 0.9	1.0 ± 0.3	<0.001
eGFR (mL/min/1.73 m^2^)	61.6 ± 26.1	82.0 ± 20.9	<0.001
Length of stay (days)	10.4 ± 13.0	6.9 ± 6.3	<0.001
Refer for hemodialysis, *n* (%)	4 (2.1)	2 (0.1)	0.003
Dead in hospital, *n* (%)	34 (17.4)	39 (2.8)	<0.001

Numbers are *n* (%), or mean ± SD. Abbreviations: AKI, acute kidney injury; eGFR, estimated glomerular filtration rate; pPCI, primary percutaneous coronary intervention.

**Table 5:** Methodological characteristics of clinical scoring systems used for comparative validation in this study.

Scoring	Country (Sample Size)	Domain	Predictors		AKI Definition
			Pre-Procedural	Intra-Procedural	
CCIT score [19]	Thailand (217)	Patients with STEMI underwent emergency pPCI	LVEF < 40%	Triple vessel disease, use of IABP	≥0.5 mg/dL or ≥25% increase in serum creatinine from baseline within 48 h of pPCI
Mehran’s risk score [24]	United States (5571)	Patients with ACS underwent PCI	Age, anemia, DM, basal creatinine, CHF	Contrast media volume, use of IABP, hypotension	≥0.5 mg/dL or ≥25% increase in serum creatinine from baseline at 48 h after PCI
Ghani&Tohamy score [25]	Kuwait (247)	Patients with ACS admitted for PCI	Female, DM, basal creatinine	Multi-vessel stenting, shock	≥0.5 mg/dL increase in serum creatinine from baseline within 48 h of PCI
AGEF score [27]	Italy (481)	Patients with STEMI underwent emergency pPCI	Age, LVEF, eGFR		increase in serum creatinine ≥0.5 mg/dL or ≥25% from baseline within 72 h
PPCI-AKI score (This study)	Thailand (1617)	Patients with STEMI underwent emergency pPCI	Age, baseline creatinine, LVEF < 40%, cardiogenic shock, CHF	Multi-vessel pPCI, use of IABP, Thrombus aspiration, cardiogenic shock, CHF	increase in serum creatinine ≥0.3 mg/dL from baseline within 48 h after pPCI

Abbreviations: ACS, acute coronary syndrome; AGEF score, age, glomerular filtration rate, ejection fraction score; AKI, acute kidney injury; CCIT, Central Chest Institute of Thailand; CHF, congestive heart failure; DM, diabetes mellitus; eGFR, estimated glomerular filtration rate; IABP, intra-aortic balloon pump; LVEF, left ventricular ejection fraction; pPCI, primary percutaneous coronary intervention; PCI, percutaneous coronary intervention; STEMI, ST-segment elevation myocardial infarction.

## Data Availability

The datasets used and/or analyzed during the current study are available from the corresponding author on reasonable request.

## Supplementary Materials

The following are available online at https://www.mdpi.com/article/10.3390/jcm10153402/s1, Table S1: Definitions of prognostic determinants, Table S2: Lists of previous medication used in the study cohort, Table S3: Sensitivity and specificity of each score cutoff point for detecting AKI after pPCI.
