# Supplementary material for: Clinical Scoring for Prediction of Acute Kidney Injury in Patients with Acute ST-Segment Elevation Myocardial Infarction after Emergency Primary Percutaneous Coronary Intervention
Source: J Clin Med. 2021 Jul 30;10(15):3402. doi: 10.3390/jcm10153402 (PMC8348987; doi:10.3390/jcm10153402)
Supplement: Supplementary file 1 [file jcm-10-03402-s001.zip › jcm-1302910-supplementary.pdf]

Supplementary Materials

Clinical Scoring for Prediction of Acute Kidney Injury in Patients with Acute ST-Segment Elevation Myocardial Infarction after Emergency Primary Percutaneous Coronary Intervention

Akaphol Kaladee [1], Phichayut Phinyo [2,3,4,*], Thamarath Chantadansuwan [5], Jayanton Patumanond [3] and Boonying Siribumrungwong [6]

1 School of Health Science, Sukhothai Thammathirat Open University, Nonthaburi 11120, Thailand; akaphol.ka@gmail.com
2 Department of Family Medicine, Faculty of Medicine, Chiang Mai University, Chiang Mai 50200, Thailand
3 Center for Clinical Epidemiology and Clinical Statistics, Faculty of Medicine, Chiang Mai University, Chiang Mai 50200, Thailand; jpatumanond@gmail.com
4 Musculoskeletal Science and Translational Research (MSTR) Center, Chiang Mai University, Chiang Mai 50200, Thailand
5 Cardiology Department, Central Chest Institute, Nonthaburi 11000, Thailand; dansuwan_t@yahoo.co.th
6 Department of Surgery, Faculty of Medicine, Thammasat University, Pathum Thani 10120, Thailand; boonying22@gmail.com
* Correspondence: phichayutphinyo@gmail.com

Supplementary Table S1. Definitions of prognostic determinants

**Determinants**	**Definitions**
Previous medication used	All previously prescribed cardiovascular-related medications, both for prevention and therapy, documented in patients' hospital records. The groups of medications collected were as follow: angiotensin converting enzyme inhibitors (ACEI) (i.e., enalapril), angiotensin receptor blocker (ARB) (i.e., losartan), diuretics (i.e., hydrochlorothiazide), calcium channel blockers (i.e., amlodipine), beta-blockers (i.e., atenolol), antiplatelet (i.e., aspirin), anti-diabetic drugs (i.e., metformin, glibenclamide, glipizide), and cholesterol-lowering drugs (i.e., statins and fibrates).
Pre- and intra-procedural hypotension	The patient had systolic blood pressure <90 mmHg or diastolic blood pressure <60 mmHg during the pre-procedural and intra-procedural period. The pre-procedural period was defined as the time since the patient's arrival to the start of pPCI. The intra-procedural period was defined as the time from the start of pPCI until the end of pPCI before the patient was transferred to the cardiac critical care unit.

Pre- and intra-procedural cardiogenic shock	The patient had systolic blood pressure <90 mmHg with symptoms/signs of hypoperfusion (e.g., cold sweated extremities, oliguria, alteration of consciousness, dizziness, narrow pulse pressure) and requiring inotropic support with medications or intra-aortic balloon pump (IABP) during the pre-procedural and intra-procedural period. The pre-procedural period was defined as the time since the patient's arrival to the start of pPCI. The intra-procedural period was defined as the time from the start of pPCI until the end of pPCI before the patient was transferred to the cardiac critical care unit.
Pre- and intra-procedural CHF	Patients diagnosed with congestive heart failure by attending physicians during pre- and intra-procedural period of pPCI. The following criterion were used during data retrieving: 1) symptoms/signs of congestion (e.g., orthopnea, paroxysmal nocturnal dyspnea, pulmonary rales or fine crepitations during auscultations), 2) radiographic signs of pulmonary congestion or pulmonary edema from chest radiographs, 3) desaturation (oxygen saturation <90-95%), and 4) documented prescription of diuretics (e.g., furosemide). The intra-procedural period was defined as the time from the start of pPCI until the end of pPCI before the patient was transferred to the cardiac critical care unit.
Total ischemic time	Time from the onset of chest pain to the time of the restoration of coronary blood flow by pPCI (in minutes). Total ischemic time encompasses two time intervals, which are onset-to-door and door-to-balloon times.

Supplementary Table S2. Lists of previous medication used in the study cohort

Medication	AKI (n=195)	No AKI (n=1,422)	p-value
	n (%)	n (%)	
Prior medication	50 (25.6)	365 (25.7)	0.536
Enalapril	10 (5.1)	99 (6.7)	0.445
Amlodipine	6 (3.1)	47 (3.3)	0.867
Losartan	10 (5.1)	46 (3.2)	0.206
Hydrochlorothiazide	4 (3.6)	30 (3.1)	0.957
Atenolol	6 (3.1)	60 (4.2)	0.564
Metformin	4 (3.6)	49 (5.0)	0.394
Glibenclamide	1 (0.9)	18 (1.8)	0.719
Glipizide	4 (2.1)	58 (4.1)	0.230
Statins	22 (11.3)	170 (12.0)	0.906
Gemfibrozil	1 (0.5)	22 (1.6)	0.348
Aspirin	13 (11.4)	71 (7.1)	0.304

Abbreviation: AKI, acute kidney injury.

Supplementary Table S3. Sensitivity and specificity of each score cutoff point for detecting AKI after pPCI.

Score cutoff point	Sensitivity	95% CI	Specificity	95% CI
≥2	89.7%	84.6% - 93.6%	41.0%	38.4% - 43.6%
≥3	81.5%	75.4% - 86.7%	56.9%	54.3% - 59.5%
≥4	68.7%	61.7% - 75.2%	70.7%	68.3% - 73.1%
≥5	55.9%	48.6% - 63.0%	82.9%	80.9% - 84.8%
≥6	49.2%	42.0% - 56.5%	88.3%	86.5% - 89.9%

Abbreviations: AKI, acute kidney injury; CI, confidence interval; pPCI, primary percutaneous coronary intervention.
